# SUBCUTANEOUS RHINOSPORIDIOSIS

**DOI:** 10.4103/0019-5154.39746

**Published:** 2008

**Authors:** Surajit Nayak, Tapas Kumar Rout, Basanti Acharjya, Manoj Kumar Patra

**Affiliations:** *From Department of Skin and VD, MKCG Medical College and Hospital, Berhampur - 760 010, Orissa, India. E-mail: surajitnyk@yahoo.co.in*; 1 *From Department of Surgery, MKCG Medical College and Hospital, Berhampur - 760 010, Orissa, India*; 2 *From Department of Pathology, MKCG Medical College and Hospital, Berhampur - 760 010, Orissa, India*

Rhinosporidiosis is a chronic granulomatous disease involving man and lower animals, caused by *Rhinosporidium seeberi*, endemic in South India and Sri Lanka. The disease, while being a common presentation for oto-rhino-laryngologists, is of interest to dermatologists as well, because of the cutaneous, subcutaneous presentation in rare cases. Cutaneous and subcutaneous lesions are infrequent and are generally associated with mucosal lesions. We report this rare case of subcutaneous rhinosporidiosis, in absence of any primary mucosal/cutaneous lesions.

**Fig. 1:**
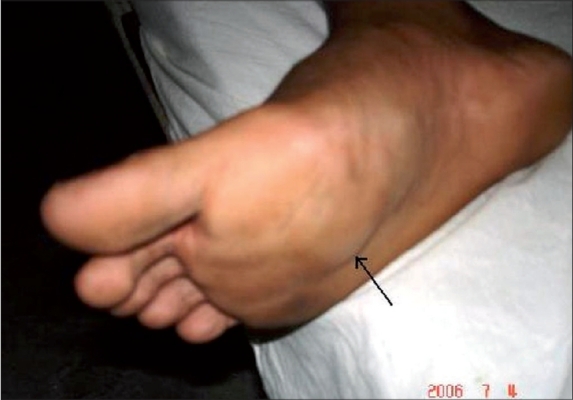
Lobular swelling

**Fig. 2:**
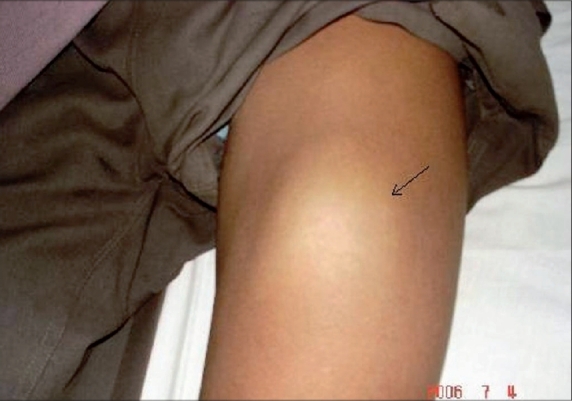
Another lesion over lower thigh

**Fig. 3:**
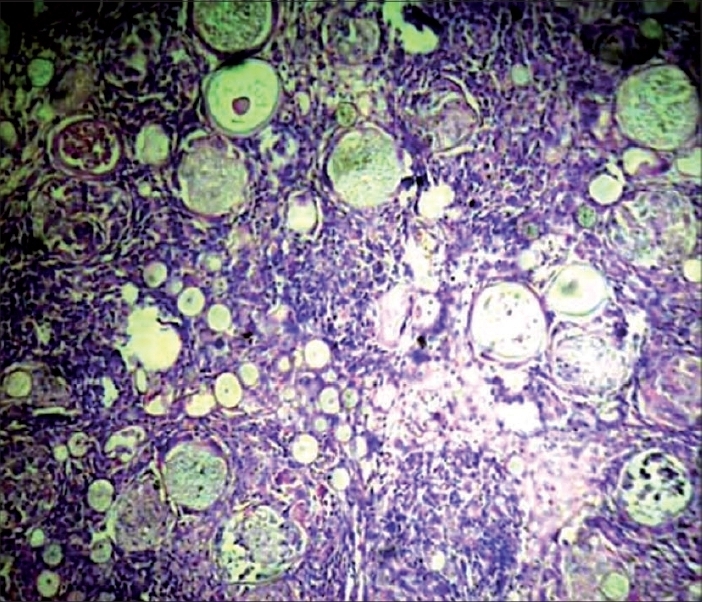
Numerous thick-walled sporangia in a vascular connective tissue along with granulomatous inflammation

**Fig. 4:**
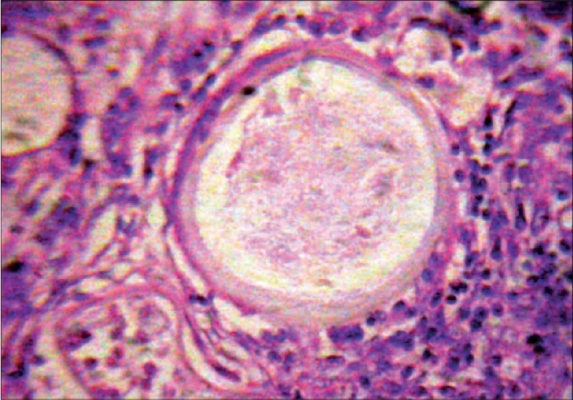
A mature sporangium in close-up view

The patient, a 23-year-old healthy agricultural labourer, presented to our clinic with multiple, asymptomatic, lobular swellings over the right foot, left thigh, abdomen and back. As narrated by the patient, it all started as a small non-tender swelling over the plantar aspect of the right foot two years back and subsequently similar lesions appeared elsewhere on the body. Patient denied any history of any trauma in the past. Examination revealed cystic swellings of different sizes, ranging from 4 cm to 8 cm over different parts as specified earlier (Fig. 1 and Fig. 2). All were non-tender, ill-defined with an overlying normal-colored skin. There was no regional lymphadenopathy and the rest of the clinical examination was normal. Routine laboratory tests and biochemical parameters were within normal limits. Patient denied any sexual contact in the recent past; VDRL and ELISA test for HIV was negative. A FNAC was advised which showed sporangia at various stages of development, subsequently confirmed by excisional biopsy (Figs. 3 and 4). A clinico-pathological diagnosis of subcutaneous rhinosporidiosis was made. An ultrasonogram and X-Ray chest excluded internal involvement. Patient was referred to surgeon for excision of individual lesions. He was discharged and was advised to take dapsone (100 mg/daily) and asked to report for regular follow-up.

Though rhinosporidiosis and the etiological agent *Rhinosporidium seeberi,* is known for over a hundred years and was first described in Argentina,^1^ the as yet unresolved enigmas in rhinosporidiosis include the mode of infection, mechanisms of spread, mechanisms of immunity, some aspects of histopathology, e.g. the significance of transepidermal elimination of sporangia, the cause of the variation in cell infiltration patterns in rhinosporidial tissues and their correlations with immune status and the absence of the Splendore-Hoeppli reaction which is well-marked in invasive, classical mycoses. Infection is usually caused by contact with stagnant fresh water such as swimming pools.*R. seeberi* had first been regarded as a sporozoan by Malbran, its discoverer, in 1892, as a protozoan by Seeber who first published a description of the pathogen and then, as a phycomycete by Ashworth in 1923.^2^ Its taxonomic position is unclear till date, but a recent isolation of a prokaryotic cyanobacterium *Microcystis aeruginosa* by Ahluwalia *et al.*^3^ from water samples of ponds and rivers where patients were bathing and detection of both large cells and nanocytes of *M. aeruginosa* inside round bodies of rhinosporidiosis by light and electron microscope has virtually ended all etiological controversies surrounding rhinosporidiosis. This new finding may reinforce the justification for a change in the name “rhinosporidiosis” that has been traditionally associated with the fungus *R. seeberi*. It is characterized by the development of pedunculated and sessile polypoid lesions on the mucosa of the nose, eyes, larynx, penis and rarely on other parts of the body like skin. Subcutaneous lesions are extremely rare and very few cases presenting only as a subcutaneous mass have been reported. It is a chronic disease, with frequent recurrence after surgery and occasional dissemination from the initial focus, that is most commonly seen in upper respiratory sites, was not observed in our case. This type of presentation needs to be differentiated from lipoma, sebaceous cyst and fibroma and disseminated pheohyphomycosis. As there are many reports of rhinosporidiosis of different types by different authors, clinical presentation is no more a mystery and I will be highlighting a little more about the etiolological agent and its unsolved enigmas in shadow of recent studies in my discussion. Rhinosporidiosis is an infective disease in the sense that the tissue lesions are always associated with the presence of the pathogen. No evidence has been adduced that it is also an infectious disease, as no transmission has ever been documented of cross-infection between members of the same family or between animals and humans. A curious feature in the incidence of the disease is that while several hundreds of persons bathe in stagnant waters, as in our country, only a few develop progressive disease; this might indicate the existence of predisposing, though obscure, factors in the host. The only patient factor on which there is some data is blood groups. In India the highest incidence of rhinosporidiosis was in Group O (70%) though in the population Groups A, B and O are distributed “fairly equally”; the next highest incidence was in Group AB though in the general population Group AB is rare.^4^ Jain,^5^ in India, reported that the blood group distribution was too variable to draw any conclusion. The presumed mode of infection from the natural aquatic habitat of *R. seeberi*, is through the traumatized epithelium (‘transepithelial infection’)^6^ most commonly in nasal sites, but there is evidence for hematogenous and lymphatic spread of rhinosporidiosis to anatomically distant sites.^7^ The development of subcutaneous granulomata in the plantar aspect of the right foot, without breach of the overlying skin, as seen in our case could be attributed to any trivial trauma not noticed by the patient and subsequent hematogenous dissemination. Autoinoculation was considered by Karunaratne^8^ in his classical monograph on rhinosporidiosis, to be the explanation for the occurrence of satellite lesions adjacent to granulomas, especially in the upper respiratory sites and for local spread. In the absence of pure preparations of the developmental stages of *R. seeberi*, uncontaminated by host tissue and other micro-organisms and consequently the absence of specific tests for immune responses in diseased hosts, it is not surprising that there was very little data, till recently, on the immune responses in rhinosporidiosis. The only report on tests for anti-rhinosporidial antibody in human patients was by Chitravel *et al.*^9^ where no antibody was detected in double diffusion or CIE tests. The CMI responses (CMIR) in human rhinosporidiosis were re-examined recently^10^ by immunohistology with monoclonal antibodies to specific cell markers on cells in rhinosporidial tissues, which showed that the cell infiltrate in human rhinosporidial polyps was mixed, having consisted of many plasma cells, fewer CD 68+ macrophages, CD 3+ and CD 56/57+ NK lymphocytes which were positive for CD 3 as well; CD 4+ lymphocytes were present though scarce. CD 8+ suppressor/cytotoxic lymphocytes were numerous and most of the CD 8+ cells were TIA-1+ and therefore of the cytotoxic subtype.

Although cases of spontaneous regression have been recorded, they are rare and the mode of treatment remains surgical. Total excision of the polyp, preferably by electro-cautery, is recommended. The failure to propagate *R. seeberi in vitro*, with the inability to establish experimental rhinosporidiosis, have prevented the determination of the sensitivity of *R. seeberi* to drugs that might have clinical application. While several anti-bacterial and anti-fungal drugs have been tested clinically, but unsuccessfully, the only drug which was found to have some anti-rhinosporidial effect is dapsone (4,4-diaminodiphenyl sulphone)^11^,^12^ which appears to arrest the maturation of the sporangia and to promote fibrosis in the stroma, when used as an adjunct to surgery.
